# Early improvement in shoulder external rotation proprioception after arthroscopic rotator cuff repair: an AMEDA-based quantitative assessment

**DOI:** 10.3389/fbioe.2026.1904367

**Published:** 2026-07-29

**Authors:** Jinfeng Cao, Chengshuo Xu, Xinyu Peng, Yangyu Chen, Jia Han, Jie Lyu

**Affiliations:** 1 Department of Orthopedics, Jinshan District Central Hospital Affiliated to Shanghai University of Medicine and Health Sciences, Shanghai, China; 2 Periodicals Agency of Shanghai University, Shanghai University, Shanghai, China; 3 College of Rehabilitation Sciences, Shanghai University of Medicine and Health Sciences, Shanghai, China; 4 Research Institute for Sport and Exercise, University of Canberra, Canberra, ACT, Australia

**Keywords:** AMEDA, arthroscopic repair, AUC, Constant–Murley score, rotator cuff injury, shoulder proprioception

## Abstract

**Objective:**

Rotator cuff injury often disrupts shoulder proprioception. However, early postoperative recovery of external rotation proprioception after arthroscopic repair lacks quantification via ecologically valid tools. This study aimed to quantify early improvement in shoulder external rotation proprioception following arthroscopic rotator cuff repair using the Active Movement Extent Discrimination Apparatus (AMEDA), and to identify preoperative factors associated with postoperative proprioceptive improvement.

**Methods:**

We included thirty-one patients with unilateral rotator cuff injury who underwent arthroscopic repair. Proprioceptive acuity was quantified as the area under the receiver operating characteristic curve (AUC). Pain (Visual Analogue Scale, VAS) and shoulder function (Constant–Murley Score, CMS) were recorded before and after surgery. The Shapiro–Wilk test was used to check normality. Paired t -tests and Pearson correlations were applied to analyze which factors influenced proprioceptive improvement ( ΔAUC , defined as postoperative AUC minus preoperative AUC).

**Results:**

Preoperatively, injured shoulders showed significantly lower proprioceptive AUC than the contralateral sides ( p<0.0001 ). After surgical repair, affected-side AUC increased markedly, becoming statistically comparable to those of contralateral sides ( p=0.1091 ). At the same time, VAS pain dropped and CMS improved (both p<0.0001 ). The proprioceptive improvement ( ΔAUC ) had a strong negative correlation with preoperative AUC ( r=−0.625 ). After Bonferroni correction, only preoperative AUC remained statistically significant.

**Conclusion:**

AMEDA enables credible, quantitative proprioception measurement for shoulder external rotation proprioception in patients with rotator cuff injury. Arthroscopic rotator cuff repair produces measurable early improvements in shoulder proprioception. A negative linear association between preoperative proprioceptive acuity and postoperative proprioceptive improvement is observed, supporting the need for personalized perioperative proprioception rehabilitation. However, longer-term follow-up is needed to confirm sustained improvement.

## 1 Introduction

Rotator cuff injury (RCI) represents one of the most common musculoskeletal problems, characterized by shoulder pain and functional impairment of the glenohumeral joint (Tytherleigh-Strong et al., 2001). The rotator cuff, as one of the body’s largest tendon structures, is essential for the movement and stability at the shoulder joint. However, the exceedingly high functional demands placed on this structure make it prone to overload and failure (Tytherleigh-Strong et al., 2001). Given the high incidence of these injuries and their significant social and economic costs, comprehensive evaluation of RCI management approaches is of paramount importance (Vieira et al., 2015).

Apart from the structural damage, RCI is intrinsically associated with proprioceptive deficits, a critical yet often overlooked consequence. Proprioception, defined as the ability to perceive and integrate the position and movement sense of body parts to determine their status in space, plays an essential role in neuromuscular control (Han et al., 2016a). The static and dynamic functions of joint stabilizers are integrated through this sensory mechanism (Lubiatowski et al., 2013). In RCI patients, impaired proprioception adversely affects joint stability and motor control, creating a vicious cycle of dysfunction. Warner et al. (1996) confirmed a close correlation between shoulder instability and poor proprioceptive acuity, highlighting the bidirectional relationship between structural pathology and sensory dysfunction.

For patients failing conservative treatment, arthroscopic rotator cuff repair has become an effective intervention to recover shoulder function (Meister and Andrews, 1993). Modern arthroscopic techniques have significantly improved recovery outcomes (Bedi, et al., 2024), enabling such procedures to be performed in outpatient settings (Tytherleigh-Strong et al., 2001). Optimal clinical outcomes rely on precise diagnosis through systematic clinical examination and auxiliary imaging evaluation (Tytherleigh-Strong et al., 2001). Additionally, preoperative proprioceptive evaluation has been proposed to assist clinical stratification and personalized treatment allocation for RCI patients (Coscia et al., 2021). Despite well-documented efficacy in restoring shoulder mechanical integrity after arthroscopic repair, a critical research question remains unresolved: whether surgical reconnection of the torn tendon could restore damaged shoulder proprioception in the early postoperative stage.

Emerging evidence in orthopedic surgery indicates that structural repair alone may be insufficient to restore neuromuscular integrity. After successful anterior cruciate ligament (ACL) reconstruction, many patients retain proprioceptive deficits that limit functional recovery (Fleming et al., 2022; Zhao et al., 2023). Anatomical repair does not guarantee sensorimotor recovery. Similarly, technically successful rotator cuff repair may leave patients with residual proprioceptive impairment, hindering their ability to fully exploit restored anatomy during dynamic tasks.

This knowledge gap carries considerable clinical implications. Prior studies have shown that postoperative proprioceptive insufficiency prevents patients from fully utilizing their restored range of motion (Kasten et al., 2009), potentially compromising long-term functional outcomes. Traditional proprioception testing equipment, such as computerized isokinetic machines, handheld dynamometers, and three-dimensional motion capture systems, is limited by poor ecological validity, extensive calibration procedures, and reliance on specialized operational expertise (Fox et al., 2024). In addition, most existing proprioception testing protocols have not been thoroughly psychometrically validated (Ager et al., 2017), limiting their clinical application.

Addressing this methodological challenge requires an assessment approach that balances precision with functional relevance. As emphasized by Han et al. (2016a), proprioceptive assessment should be ecologically valid and tested in normal functional circumstances. Building on this principle and recognizing the shared neural mechanisms of proprioception across joints, Han et al. (2016a) developed the Active Movement Extent Discrimination Apparatus (AMEDA). The AMEDA captures somatosensory sensitivity during active movement, offering superior ecological validity compared to passive or static testing methods (Han et al., 2016a, Han et al., 2016b). This approach aligns with broader trends in sensorimotor assessment, as evidenced by Shao et al. (2022), who developed an apparatus for assessing ankle proprioception during walking and demonstrated its clinical relevance to fall risk in older adults. Subsequent research has further reinforced the importance of functional testing by demonstrating that proprioceptive impairments may be task-specific (Han et al., 2022).

Accordingly, we adopted AMEDA to quantify early postoperative external rotation proprioceptive changes and identify preoperative variables associated with sensory improvement after arthroscopic rotator cuff repair.

## 2 Methods

### 2.1 Participants

This study recruited patients with unilateral partial-thickness rotator cuff injury from Jinshan District Central Hospital Affiliated to Shanghai University of Medicine and Health Sciences. All patients with partial-thickness rotator cuff injuries in this study were diagnosed by standardized shoulder physical examination combined with 3.0T shoulder MRI screening, and intraoperative arthroscopy served as the gold standard to confirm partial rotator cuff tendon lesions and rule out complete tendon rupture. Eligible participants were aged 40–75 years and provided written informed consent prior to enrollment. Exclusion criteria were as follows: (1) concomitant fractures around the shoulder girdle; (2) severe cardiovascular diseases (e.g., essential hypertension), other systemic disorders, psychiatric diseases, or cognitive impairment; (3) inability to tolerate or complete proprioception testing; (4) patients with bilateral rotator cuff injury. An *a priori* power analysis confirmed that the sample size of 31 patients included in this study provided adequate statistical power for the primary paired comparison.

Shoulder function was evaluated by trained assessors using the Constant–Murley Score (CMS). The CMS is a validated 100-point shoulder assessment instrument comprising objective clinician-measured parameters and subjective patient-reported outcomes, which is widely adopted for functional evaluation in patients with rotator cuff lesions (Roy et al., 2010).

This study was performed in accordance with the Declaration of Helsinki and approved by the Medical Ethics Committee of Jinshan District Central Hospital Affiliated to Shanghai University of Medicine and Health Sciences (Approval No. ZFKY202505).

### 2.2 Apparatus

Shoulder proprioceptive acuity was measured using the modified AMEDA. This device delivers high ecological validity for shoulder proprioception testing (Huang et al., 2024). Developed based on signal-detection theory, the AMEDA analyzes discrimination performance.

**FIGURE 1:**
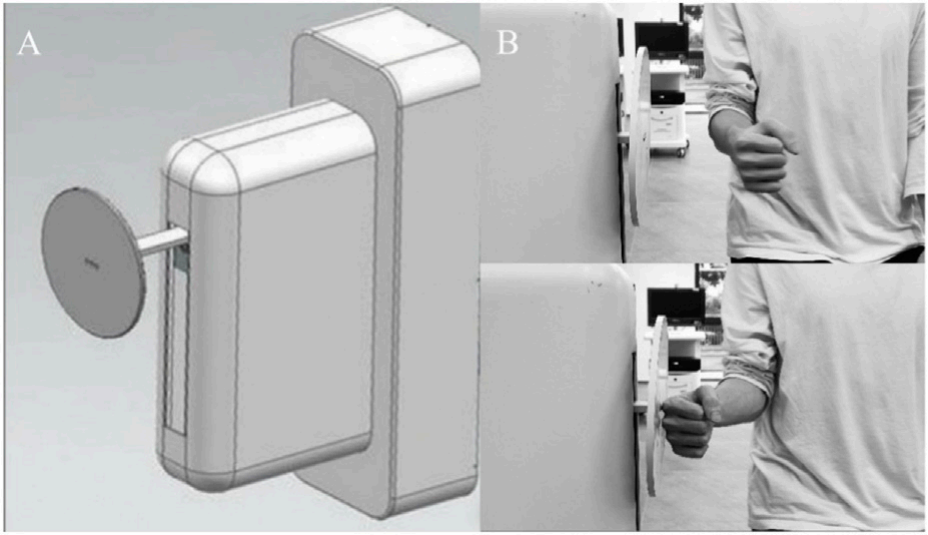
**(A)** Schematic diagram of the Active Movement Extent Discrimination Apparatus (AMEDA); **(B)** Protocol for shoulder external rotation proprioception assessment.

As shown in Figure 1A, the AMEDA consists of a fixed wall-mounted base and an adjustable-height circular disk with a radius of 10 cm. The disk is driven by two independent servo motors: one motor dynamically adjusts the vertical height of the disk to match the individual palm position of each participant, ensuring consistent and comfortable testing posture. The other motor generates four discrete graded positions corresponding to four distinct shoulder external rotation angles for proprioceptive discrimination. The base is connected to a computer that enables real-time position control and data recording. During testing, participants stood facing the apparatus with eyes open, and the tested arm was aligned with the center of the movable disk.

### 2.3 Testing procedure

The measurements were conducted preoperatively and 1 week after surgery. The testing order for the affected and unaffected shoulder was randomized among participants to minimize potential sequence-dependent testing effects. Proprioception testing included a familiarization phase and a formal testing phase. During familiarization, participants performed 12 practice trials of shoulder external rotation to experience 4 target positions in ascending order (from nearest to farthest), with each position presented 3 times.

In the formal test, the position of the movable disk was randomized. Participants were instructed to verbally classify the perceived magnitude of shoulder external rotation into 4 graded levels (numbered 1–4). After each trial, participants could proceed immediately or rest for up to 1 min. A total of 40 trials were administered, with each of the 4 positions presented 10 times in random order; the total testing duration was approximately 20 min per participant. One trained investigator controlled the mechanical stops and recorded all judgments. No feedback regarding actual position was provided during formal testing (Figure 1B).

### 2.4 Postoperative rehabilitation protocol

All patients followed a standardized early rehabilitation protocol within the first postoperative week, with no additional shoulder proprioception or strength training. The affected shoulder was fully immobilized in an abduction brace. Only gentle distal limb and scapular movements were allowed, including active finger and wrist motions, elbow flexion-extension, and mild scapular retraction and depression. Any active shoulder elevation, external rotation, or internal rotation beyond the brace neutral position was forbidden.

These minimal exercises were designed to promote peripheral circulation and prevent soft tissue adhesion. No specialized neuromuscular or proprioceptive training was provided during the first week, ensuring consistent rehabilitation conditions for all participants and minimizing confounding effects of rehabilitation on changes in proprioceptive acuity.

### 2.5 Data analysis

All statistical analyses were performed using Python (version 3.9), with statistical significance set at p < 0.05 . Proprioceptive acuity was quantified by the area under the receiver operating characteristic curve (AUC). An AUC value of 0.5 indicates that position judgements were made randomly, whereas an AUC values of 1.0 signifies perfect position discrimination. ΔAUC , defined as postoperative AUC minus preoperative AUC, was calculated to reflect changes in proprioceptive acuity. The maximum pain experienced in daily activities was assessed using a Visual Analogue Scale (VAS) (Price et al., 1983), ranging from 0 points (no pain at all) to 10 points (unbearable maximal pain). Shoulder function was evaluated via the Constant–Murley scale (CMS) (Constant and Murley, 1987). The CMS covers four subdomains including pain, daily living activities, shoulder range of motion, and muscle strength, with a total score ranging from 0 to 100; higher total scores indicate better overall shoulder functional recovery.

Prior to pairwise comparison, the Shapiro–Wilk test was used to verify the normality of differences for each paired dataset. Paired-sample *t*-tests were applied when the difference satisfied normal distribution; otherwise, the Wilcoxon signed-rank test was adopted for non-normal paired data. Three pairwise comparisons of proprioceptive AUC were performed, including preoperative affected shoulder versus the contralateral unaffected shoulder, preoperative affected shoulder versus postoperative affected shoulder, and postoperative affected shoulder versus the contralateral unaffected shoulder. Preoperative and postoperative VAS and CMS scores of the affected shoulder were also subjected to the above paired comparison strategy.

Bivariate Pearson correlation analysis was conducted to assess linear associations between ΔAUC and preoperative baseline variables, including preoperative affected AUC, preoperative VAS, preoperative CMS, and patient age. Postoperative indicators were excluded from predictive correlation analysis due to chronological bias. Normality and linearity assumptions required for Pearson correlation were validated before calculation. Bonferroni correction was used to adjust the significance threshold for multiple correlation comparisons.

## 3 Results

### 3.1 Baseline demographic characteristics

A total of 31 patients were enrolled in this study, consisting of 9 males (29.0%) and 22 females (71.0%), as shown in Table 1. Thirteen patients suffered left-sided rotator cuff injury (41.9%) and 18 patients had right-sided lesions (58.1%). The mean age of participants was 56.06±10.56 years.

**TABLE 1:** Baseline demographic characteristics of included patients.

Variables	Category	Value
Sex	Male	9 (29.0%)
​	Female	22 (71.0%)
Affected shoulder side	Left	13 (41.9%)
​	Right	18 (58.1%)
Age, years	Mean±SD	56.06±10.56

### 3.2 Descriptive statistics of outcome indicators

The distribution of proprioceptive AUC, VAS pain score, and CMS functional score measured preoperatively and postoperatively is presented in Table 2. Normally distributed indicators are expressed as mean ± standard deviation, and the non-normally distributed unaffected-side AUC is reported as median (interquartile range, IQR). Compared with preoperative affected-side AUC ( 0.736±0.075 ), postoperative affected-side AUC increased to ( 0.781±0.059 ), which was close to the level of the contralateral unaffected side (median: 0.807, interquartile range: 0.770–0.847). Postoperative VAS decreased distinctly, whereas CMS scores increased significantly after surgery.

**TABLE 2:** Descriptive statistics of AUC, VAS, and CMS at different time points.

Indicator	Statistical value
Unaffected-side AUC	Median (IQR): 0.807 (0.770–0.847)
Preoperative affected AUC	Mean±SD:0.736±0.075
Postoperative affected AUC	Mean±SD:0.781±0.059
Preoperative VAS	Mean±SD:5.242±0.542
Postoperative VAS	Mean±SD:3.126±0.545
Preoperative CMS	Mean±SD:63.129±3.594
Postoperative CMS	Mean±SD:71.161±4.458

### 3.3 Intraindividual paired comparisons

Detailed results of all five paired comparisons are listed in Table 3. Preoperative affected-side AUC was significantly lower than the contralateral unaffected AUC ( p<0.0001 ). Affected shoulder AUC improved significantly after surgical repair ( p<0.0001 ), and no significant between-side discrepancy existed between postoperative affected AUC and contralateral unaffected AUC ( p=0.1091 ). Postoperative pain and shoulder function were both significantly improved relative to preoperative status (both p<0.0001 ).

**TABLE 3:** Results of paired comparison.

Paired comparison	Normality	Test method	Mean/Median difference	95% CI/IQR	Test statistic	p	Effect size
Unaffected AUC vs. pre-op affected AUC	0.0041	Wilcoxon signed-rank	−0.063	[−0.087, −0.017]	Z=−4.380	<0.0001	r=0.787
Unaffected AUC vs. post-op affected AUC	0.5482	Paired-sample t -test	−0.018	[−0.041, 0.004]	t=−1.652	0.1091	d=−0.297
Pre-op affected AUC vs. post-op affected AUC	0.5403	Paired-sample t -test	0.046	[0.025, 0.066]	t=4.508	<0.0001	d=0.810
Pre-op VAS vs. post-op VAS	0.2458	Paired-sample t -test	−2.116	[−2.197, −2.036]	t=−53.742	<0.0001	d=−9.652
Pre-op CMS vs. post-op CMS	0.0441	Wilcoxon signed-rank	8.000	[6.000, 10.000]	Z=−4.860	<0.0001	r=0.873

* For paired t -test, difference = post value − pre value; effect size Cohen’s d = mean difference/standard deviation of differences. For Wilcoxon test, median difference is used, effect size $r=\left|Z\right|/\sqrt{n}$ , where n is the total number of patients.

### 3.4 Bivariate Pearson correlation between ΔAUC and preoperative variables

All included variable pairs satisfied the prerequisites for Pearson correlation analysis, and relevant correlation results are summarized in Table 4.

**TABLE 4:** Pearson correlation between ΔAUC and preoperative clinical variables.

Variable pairs	Pearson r	Raw p	Significance (uncorrected)	Correlation description
ΔAUC vs. age	0.0508	0.7859	Not significant	Near-zero linear correlation
ΔAUC vs. preoperative AUC	−0.6250	0.0002	Highly significant	Moderate-to-strong negative correlation
ΔAUC vs. preoperative VAS	0.4419	0.0128	Significant	Moderate positive correlation
ΔAUC vs. preoperative CMS	−0.3670	0.0423	Significant	Weak-to-moderate negative correlation

Raw p= unadjusted p -value; after Bonferroni correction ( α=0.0125 ), only preoperative AUC remained statistically significant.

No significant linear correlation was detected between patient age and ΔAUC ( r=0.0508 ; p=0.7859 ). A strong negative correlation was found between preoperative affected-side AUC and ΔAUC ( r=−0.625 ; p=0.0002 ), which is consistent with limited gain in high-functioning patients, although this association is subject to statistical biases detailed in the study limitations. Preoperative VAS pain score was moderately positively correlated with ΔAUC ( r=0.4419 ; p=0.0128 ), such that greater preoperative pain corresponded to larger postoperative increases in AUC. In addition, preoperative CMS was marginally negatively associated with ΔAUC at a weak-to-moderate level ( r=−0.367 ; p=0.0423 ).

After Bonferroni correction for multiple comparisons ( α=0.0125 ), only preoperative AUC remained statistically significant; preoperative VAS reached borderline significance, and preoperative CMS failed to maintain statistical significance.

## 4 Discussion

Proprioception is essential for neuromuscular control, joint stability, and daily movement (Lephart et al., 1998). The rotator cuff and surrounding soft tissues contain abundant mechanoreceptors that transmit afferent sensory signals. Any impairment to these tissues can directly compromise afferent sensory input, thereby disturbing neuromuscular regulation and shoulder motor function (Grigg, 1994; Myers et al., 1999). Consistent with these anatomical findings, our data confirmed significantly lower preoperative AUC on injured shoulders compared with contralateral healthy sides. Several factors contribute to this sensory decline. First, tendon tears directly damage peripheral sensory receptors and block neural conduction. Second, persistent pain and disuse alter central sensory processing and further reduce proprioceptive acuity. Third, patients tend to adopt compensatory movement to avoid pain, increasing the sensitivity in joint position judgment. A previous study focusing on force perception, another key component of proprioception, also identified sensory abnormalities in rotator cuff patients (Maenhout et al., 2012).

Arthroscopic rotator cuff repair effectively improved patients’ proprioceptive ability, accompanied by pain relief and functional recovery. Surgical repair restores the continuity of the rotator cuff structure, which may facilitate conditions for sensory recovery. In addition, surgery effectively alleviates pain and inflammation, removing the peripheral and central inhibitory effects on proprioceptive processing. It should be noted that pain relief may partially boost proprioceptive test performance. However, the correlation between preoperative pain and proprioceptive gains lost significance after Bonferroni correction, indicating that the improved AUC cannot be fully attributed to pain reduction alone. Postoperative rehabilitation further enhances muscle strength, motor control, and neuromuscular coordination, which synergistically promote proprioceptive recovery (Fox et al., 2024). This improvement is consistent with previous studies that reported restored joint position sense following arthroscopic procedures for shoulder instability or other joint injuries (Barrett et al., 1991; Machner et al., 1998).

Beyond peripheral structural and nociceptive relief, adaptive reorganization of sensorimotor brain regions also accounts for recovered joint position discrimination. Chronic rotator cuff injuries trigger structural and functional remodeling of the primary somatosensory and motor cortices, alongside disrupted corticospinal signal transmission and defective multisensory integration (Berth et al., 2009; Conboy et al., 2021). By eliminating sustained inflammatory nociceptive input, arthroscopic surgery reverses aberrant cortical activity and recovers normal corticomuscular coupling (Chu et al., 2026). The elevated AUC values observed in our study are jointly driven by restored peripheral afferent signals and adaptive central neural reorganization. Longitudinal research combining synchronous EEG-sEMG or fNIRS-sEMG multimodal recordings (Chen et al., 2026) may help quantify postoperative corticomuscular coupling and elaborate this neural mechanism in greater detail.

We observed clear differences in postoperative proprioceptive gains across participants. No uniform reference threshold has been established for AMEDA AUC changes. Nevertheless, the observed AUC increase in this study carries clinical meaning. It eliminated preoperative between-side proprioceptive asymmetry and was accompanied by marked pain relief and functional improvement, indicating recovery of shoulder function. Correlation analysis revealed a strong negative association between preoperative proprioceptive level and postoperative improvement, and this result remained reliable after Bonferroni correction. Patients with higher baseline proprioception showed less absolute gain, possibly due to a smaller potential for improvement or regression to the mean. However, this correlation is also affected by mathematical coupling originating from the calculation of ΔAUC **.** Preoperative pain and shoulder function showed correlations with proprioceptive improvement before statistical adjustment, yet these associations disappeared after correction. This suggests that pain and overall shoulder function are not independent influencing factors for sensory recovery. Additionally, patients with severe preoperative pain tended to experience greater sensory recovery. A plausible explanation is that intense pain strongly interferes with sensory processing, and pain relief after surgery leads to a distinct rebound in proprioceptive function.

From a clinical perspective, these results provide meaningful implications for postoperative rehabilitation strategies in patients with RCI. Proprioceptive function should be regarded as a key objective indicator in perioperative evaluation, rather than focusing merely on pain relief, range of motion, and muscle strength. Early initiation of proprioceptive training is recommended to facilitate sensory relearning and optimize neuromuscular control, which may help accelerate functional recovery and reduce the risk of re-injury (Anderson and Wee, 2011). Moreover, given that proprioception in daily functional activities relies on the integrated processing of both position and movement cues, rehabilitation protocols should emphasize task-specific and functional movements instead of simple joint position reproduction (Han et al., 2016a, 2022). It is also noteworthy that the contralateral asymptomatic shoulder may present compensatory changes or even latent pathological progression in patients with unilateral RCI (Pötzl et al., 2004; Kasten et al., 2009); therefore, bilateral assessment and balanced training may be necessary to avoid asymmetric overloading.

In this study, shoulder proprioception was quantitatively evaluated using the AMEDA, which is characterized by high ecological validity for assessing proprioceptive function during functional movements (Shi et al., 2023). Compared with traditional joint position sense testing, AMEDA better simulates actual proprioceptive processing in daily activities, thus improving the clinical applicability of the measured outcomes. Its assessment paradigm can be adapted to home training protocols, for example, graded external rotation discrimination with feedback: patients perform multi-level shoulder external rotation tasks at home and receive prompt feedback to refine proprioceptive perception, with training difficulty increased progressively.

This study has several limitations. First, all participants had partial-thickness tears, yet tear size data were not collected, and participants were not stratified by involved tendons, fatty degeneration, or muscle atrophy, which may introduce confounding bias. Second, this study lacked a truly healthy control group. The contralateral shoulder cannot serve that role, because asymptomatic rotator cuff pathology is common in this age group, and the high prevalence of bilateral RCI may further bias estimation of proprioceptive performance. Future studies should recruit independent healthy controls to facilitate cross-population comparisons. Third, cognitive function was not formally assessed prior to enrollment. Since cognitive status influences performance on sensory discrimination tasks, future studies may adopt the Mini-Mental State Examination (MSSE) as an inclusion criterion to standardize participants’ cognitive level. Fourth, repeated testing using the same apparatus within a one-week may have introduced a learning effect, which could interfere with the comparison of preoperative and postoperative results. Standardized pre-test familiarization trials were implemented before every assessment to reduce practice-induced performance shifts within each testing session. As established by Yang et al. (2020), this familiarization protocol can effectively control learning effects arising from repeated measurements separated by a short interval. Nevertheless, Witchalls et al. (2014) confirmed that AUC increases can still be observed during multi-day retests even with consistent pre-test familiarization at each measurement. This suggests that the postoperative elevation in AUC scores in our study may stem partly from inter-session task learning. For future trials, extended multi-block familiarization training at baseline is suggested to reduce such confounding learning effects. Fifth, the uneven sex distribution in the study sample limited the ability to explore potential sex-related differences in proprioceptive function. Existing studies (Monteleone et al., 2024) have confirmed that sex affects pain perception and proprioceptive recovery after rotator cuff injury, and future balanced gender cohorts are needed to clarify sex-specific recovery patterns. Sixth, only short-term observations at postoperative week 1 were conducted. Assessments at this early time point are disturbed by residual pain, local inflammation, and temporary neuromuscular dysfunction, which prevents us from drawing firm conclusions about full proprioceptive recovery. Long-term follow-up measurements are required to clarify sustained proprioceptive changes. Seventh, the negative correlation between preoperative AUC and ΔAUC may stem from mathematical coupling and regression-to-the-mean bias. This inherent dependency generates statistical artefacts in our single-group pre-post design. Accordingly, this association should only be viewed as a descriptive trend, not a valid predictive or biological relationship. Future work with matched controls and repeated baseline assessments can mitigate these biases and clarify the true relationship between baseline proprioception and postoperative gains.

These limitations indicate directions for further research. Future studies may include healthy controls, classify patients based on tear size, chronicity, and laterality, and adopt a long-term longitudinal design to clarify the dynamic changes in proprioception after surgery. In addition, the efficacy of standardized proprioception-oriented rehabilitation protocols deserves to be verified by randomized controlled trials, to provide more evidence-based guidance for clinical practice.

## 5 Conclusion

AMEDA provides a reliable, ecologically valid measure of shoulder external rotation proprioception in RCI patients. One week after arthroscopic repair, proprioceptive acuity improved significantly, reaching levels comparable to the contralateral side, alongside reductions in pain and improvements in function. However, the absence of a healthy control group and long-term follow-up limits causal inference. A negative linear association between preoperative proprioceptive acuity and postoperative proprioceptive improvement is observed. These findings support the inclusion of preoperative proprioceptive assessment in surgical planning, but randomized controlled trials are needed to determine whether early sensory gains translate into sustained functional benefits. Furthermore, longer follow-up is necessary to confirm persistent improvements in shoulder proprioception.

## Data Availability

The raw data supporting the conclusions of this article will be made available by the authors, without undue reservation.
